# Impact of a multi-disease integrated screening and diagnostic model for COVID-19, TB, and HIV in Lesotho

**DOI:** 10.1371/journal.pgph.0001488

**Published:** 2023-08-02

**Authors:** Bulemba Katende, Moniek Bresser, Mashaete Kamele, Lebohang Chere, Mosa Tlahali, Rahel Milena Erhardt, Josephine Muhairwe, Irene Ayakaka, Tracy R. Glass, Morten Ruhwald, Bram van Ginneken, Keelin Murphy, Margaretha de Vos, Alain Amstutz, Mathabo Mareka, Sekhele Matabo Mooko, Klaus Reither, Lucia González Fernández

**Affiliations:** 1 SolidarMed, Partnerships for Health, Maseru, Lesotho; 2 Swiss Tropical and Public Health Institute, Allschwil, Switzerland; 3 University of Basel, Basel, Switzerland; 4 Butha-Buthe District Health Management Team, Butha-Buthe, Ministry of Health Lesotho, Maseru, Lesotho; 5 Mokhotlong District Health Management Team, Mokhotlong, Ministry of Health Lesotho, Maseru, Lesotho; 6 FIND, the Global Alliance for Diagnostics, Geneva, Switzerland; 7 Diagnostic Image Analysis Group, Radboud UMC, Nijmegen, The Netherlands; 8 CLEAR Methods Center, Division of Clinical Epidemiology, Department of Clinical Research, University Hospital Basel, University of Basel, Basel, Switzerland; 9 National Reference Laboratory, Ministry of Health of Lesotho, Maseru, Lesotho; 10 Division Clinical Epidemiology, Department of Clinical Research, University Hospital Basel, University of Basel, Basel, Switzerland; 11 SolidarMed, Partnerships for Health, Lucerne, Switzerland; Fundacao Oswaldo Cruz, BRAZIL

## Abstract

The surge of the COVID-19 pandemic challenged health services globally, and in Lesotho, the HIV and tuberculosis (TB) services were similarly affected. Integrated, multi-disease diagnostic services were proposed solutions to mitigate these disruptions. We describe and evaluate the effect of an integrated, hospital-based COVID-19, TB and HIV screening and diagnostic model in two rural districts in Lesotho, during the period between December 2020 and August 2022. Adults, hospital staff, and children above 5 years attending two hospitals were pre-screened for COVID-19 and TB symptoms. After a positive pre-screening, participants were offered to enroll in a service model that included clinical evaluation, chest radiography, SARS-CoV-2, TB, and HIV testing. Participants diagnosed with COVID-19, TB, or HIV were contacted after 28 days to evaluate their health status and linkage to HIV and/or TB care services. Of the 179160 participants pre-screened, 6623(3.7%) pre-screened positive, and 4371(66%) were enrolled in this service model. Of the total 458 diagnoses, only 17 happened in children. One positive rapid antigen test for SARS-CoV-2 was found per 11 participants enrolled, one Xpert-positive TB case was diagnosed per 85 people enrolled, and 1 new HIV diagnosis was done per 182 people enrolled. Of the 321(82.9%) participants contacted after 28 days of diagnosis, 304(94.7%) reported to be healthy. Of the individuals that were newly diagnosed with HIV or TB, 18/24(75.0%) and 46/51(90.1%) started treatment within 28 days of the diagnosis. This screening and diagnostic model successfully maintained same-day, integrated COVID-19, TB, and HIV testing services, despite frequent disruptions caused by the surge of COVID-19 waves, healthcare seeking patterns, and the volatile context (social measures, travel restrictions, population lockdowns). There were positive effects in avoiding diagnostic delays and ensuring linkage to services, however, diagnostic yields for adults and children were low. To inform future preparedness plans, research will need to identify essential health interventions and how to optimize them along each phase of the emergency response.

## Introduction

The spread of the severe acute respiratory syndrome coronavirus 2 (SARS-CoV-2) and the coronavirus disease 2019 (COVID-19) pandemic negatively impacted health services delivery in most countries, even where health systems were thought to be well-established [1–4]. While the pandemic created a significant increase in morbidity and mortality globally, health facilities across the globe shut down or restricted their activity to the provision of essential services. Factors such as infection control risks, inadequate staffing and lack of personal protective equipment [5] led to a substantial reduction in access to primary health care worldwide [6]. In countries with high TB and HIV prevalence, the COVID-19 pandemic reversed years of progress in the control of these conditions [7, 8].

Although the African continent reported relatively low number of COVID-19 cases, compared to other regions in the world, the impact of the pandemic on health service delivery was still very significant. Access to health facility–based services was interrupted as a result of 1) health workers shortages due to illness, self-quarantine, or strikes, 2) health facilities repurposed for COVID-19 treatment, covering minimum services, or closed completely, and 3) clients’ reluctance to attend to visits, due to fear of increased risk of COVID-19 exposure, or decreased availability of public transportation [9–11]. A review by Gizachew A. Tessema *et al*. reported substantial reduction in access to essential and general health services across the African continent [12], and in South Africa, a study in the KwaZulu Natal province reported an estimated 47.6% and 46.2% decrease in HIV testing and anti-retroviral therapy initiation respectively [13].

Lesotho is a country in the sub-Saharan region with over 2 million population [14]. In 2021 the estimated TB incidence sat at 614 per 100,000, the HIV prevalence among adults aged 15–49 years old was 20.9%, and there was approximately 55% TB/HIV coinfection [15–17]. The COVID-19 epidemic profoundly affected the health system in the country [18–22]. The first COVID-19 case was reported in May 2020, and up to December 2022, approximately 34,490 COVID-19 cases and 706 COVID-19 related deaths have been recorded. Although cases were recorded continuously since May 2020, three significant waves were described, with high incident rates in December 2020, June to September 2021, and December 2021 [23]. To reinforce the efforts against the spread of SARS-CoV-2, the government of Lesotho set up a public health strategy that regulated social norms to ensure physical distance between people. A COVID-19 Risk Determination and Mitigation Framework was created as a 5-colour scale, regulating daily life activities, depending on the COVID-19 reproductive number (R0) determined regularly. The scale of colors varied from green, blue, purple, yellow, and red. The Green color was associated with R0 <1 and the red color was associated with R0 > 2.5. Typically, at each subsequent level, social measures became more stringent, and included mandates, such as staying home, avoiding crowds, or closure of businesses and borders [24–28].

In this context, the project named *Mitigation strategies for communities with COVID-19 in Lesotho*: *MISTRAL* [29] was conceived as an initiative to expand COVID-19 diagnosis and integrate TB and HIV testing in the districts of Butha-Buthe and Mokhotlong, situated in the northeast part of the country. From December 2020 to August 2022, MistraL reinforced the health system response (Table 1), contributing to aspects such as: 1) support to the COVID-19 response coordination at district and national level; 2) addition of resources to improve health facilities infrastructure and provision of medical equipment; 3) integration of COVID-19/TB/HIV testing and linkage model; 4) addition and training of health workers for provision of services; and 5) implementation of research activities, including SARS-CoV-2 Rapid Antigen Diagnostic Tests (RDTs) validation in nasopharyngeal and nasal samples, or use and validation of a computer- aided diagnostic software for COVID-19 and TB (CAD4COVID and CAD4TB version 6; Delft Imaging Systems, the Netherlands) on chest radiography.

**Table 1:** Five intervention areas of the MistraL project in Lesotho, from December 2020 to August 2022.

	Intervention
**Coordination of COVID-19 response at district and national level**	• Participation in the COVID-19 district response through the District Health Management Teams • Participation in hospitals Infection Control Committees • Participation in national level technical working groups
**Support to health facilities infrastructure and medical equipment**	• Building well-ventilated COVID-19 screening centers at the entry of the hospitals • Reorganization of patient flow to access hospital services. • Design and printing TB-COVID-19 screening registers • Support procurement and distribution of medical equipment: protection equipment, oxygen supply (oxygen regulators, masks), dexamethasone for inpatient care • Installation of mobile digital 3 x-ray units in 3 hospitals, using CAD4TB software
**Integration of TB/COVID-19/HIV testing and linkage model**	• Case finding, triage and contacts investigation: ○ COVID-19 Pre-screening for all individuals visiting the hospitals (patients and visitors) ○ Parallel COVID-19 testing using SARS-CoV-2 rapid tests (nasal and nasopharyngeal samples), and PCR ○ TB symptoms screening and testing using Xpert Ultra • HIV testing and counselling for participants with unknown status • Linkage to TB, HIV and COVID-19 care and case management according to national guidelines of newly diagnosed patients
**Support to human resources for health**	• Capacity building, training for health workers at facility level • Health workforce deployment for key positions: doctors, screeners, nurses, laboratory technicians, radiology assistants
**Implementation of clinical research initiatives**	• Field validation a SARS-CoV-2 RDT using nasal and nasopharyngeal samples against PCR • Evaluation of an aerosol capture device for detection SARS-CoV-2 in breath • Performance measurement of CAD4COVID digital x-ray software • Platform to roll out COVID-19 rapid testing at community level • Qualitative research investigating barriers to COVID-19 testing and attitudes towards COVID-19 vaccines

In recent years, there has been an increasing interest to understand whether and how integrated screening and testing services could provide access to prompt COVID-19 diagnosis and management, while maintaining diagnostic and linkage services for TB and HIV, in an epidemic context [11, 30, 31]. Nonetheless, to date, there have been few studies demonstrating the feasibility, diagnostic yields, or wider impact of such services in sub-Saharan Africa [32–34]. The objective of this study is to describe and assess the effect of a pragmatic COVID-19/TB and HIV integrated screening and testing service model for adults and children older than 5 years in two remote referral hospitals in Lesotho.

## Methods

### Context

We implemented this screening and diagnostic model at the St Charles Missionary Hospital Seboche and the Mokhotlong Government District Hospital, in the districts of Butha-Buthe and Mokhotlong. These districts are characterized by mostly rural settings with an estimated combined population of 250,000 people, who are mainly subsistence farmers, mine workers or construction and domestic laborers who work in neighboring South Africa. Each district has only one central mid-size town: Butha-Buthe with ca. 25,000 inhabitants and Mokhotlong with ca. 10,000 inhabitants. The remaining population lives in villages scattered over a mountainous area of 5,842 km^2^. Health services in these two districts are provided through a network of clinics and hospitals. In Butha-Buthe district health care is available through ten nurse-led rural health centers, one missionary hospital, and one governmental hospital. In Mokhotlong services are available through nine nurse-led rural health centers, and one governmental hospital.

### Integrated TB/COVID-19 and HIV screening and testing model

**Fig 1:**
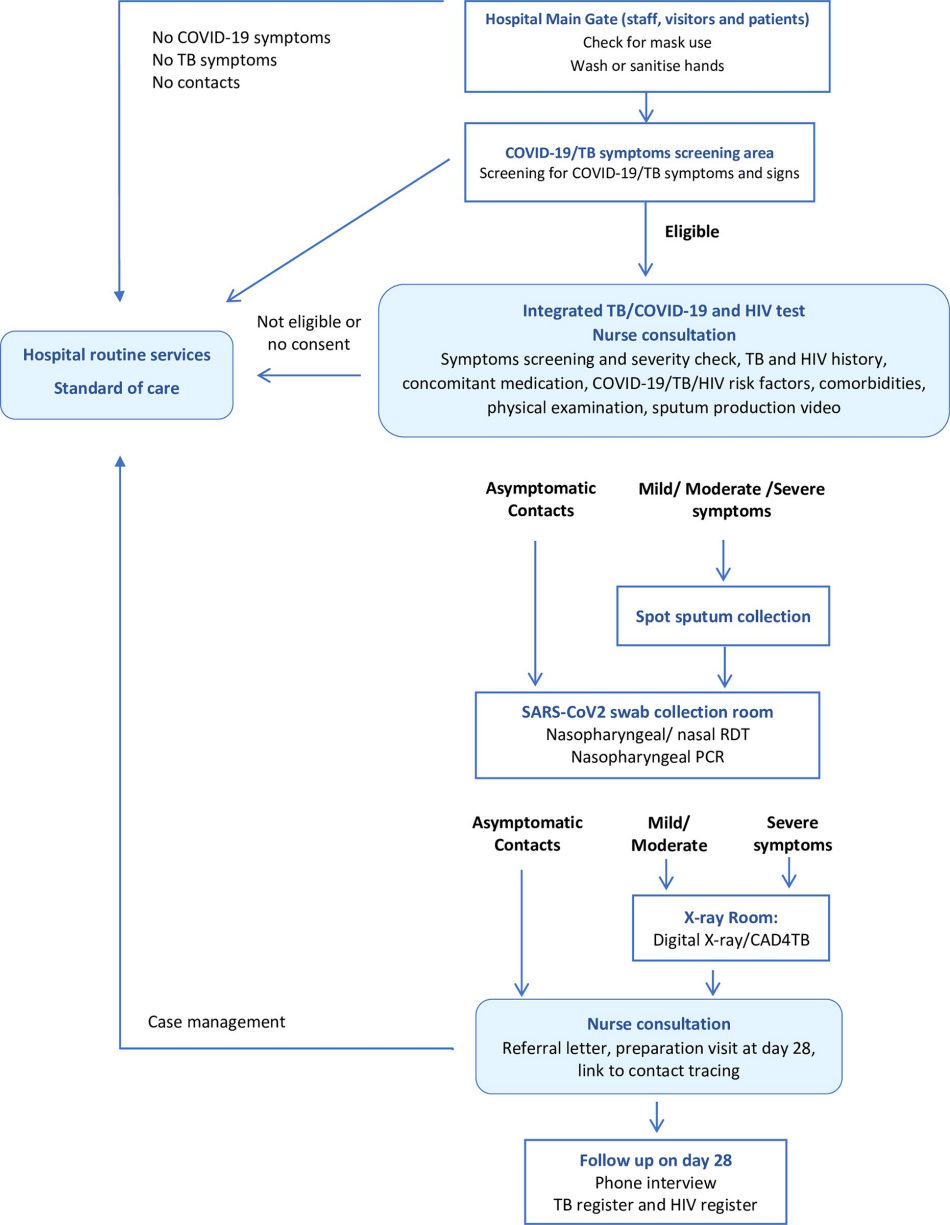
Clinical procedures in the MistraL project: Integrated, bi-directional COVID-19/TB and HIV screening and testing.

Prior to the COVID-19 epidemic, HIV and TB services were typically available at the study hospitals. All newly diagnosed TB individuals were tested for HIV by the nurses providing TB services. Similarly, individuals living with HIV followed TB investigations triggered by a positive symptoms screening performed by the nurse providing HIV care. As part of the COVID-19 response at the study sites, a nurse-led, multi-disease, integrated screening, and diagnostic model was established, with the aim to provide same-day, one-stop shop diagnosis and clinical evaluation for the three conditions to adults, children and staff attending these two hospitals. In addition to TB, COVID-19 and HIV screening and testing, nurses also systematically measured blood pressure (BP) and body mass index (BMI). After the diagnostic procedures, the nurses would manage cases themselves, or refer to other hospital staff, according to established clinical algorithms. Tests results were available to the participants on the same day (Fig 1). This service was continuously adapted to the needs and diagnostic standards, driven by a fast-changing context.

#### Screening, eligibility, and enrolment

Adults and children seeking health services, visitors, and staff attending daily work, were pre-screened by trained TB/COVID-19 lay screeners. Pre-screening took place in specially built structures by the gates of the two hospitals and focused on the investigation of history of close contact with a COVID-19 case or the presence of at least one sign or symptom of TB or COVID-19. The initial evaluation included: fever measured (≥38°C, forehead or reported), cough of any duration, pronounced tiredness, shortness of breath, sore throat, muscle or body pain, diarrhoea, loss of taste/smell, weight loss, and night sweats. We defined close contact with a (confirmed or probable) COVID-19 case, as contact within one meter for more than 15 minutes, or direct physical contact with a (probable or confirmed) case, or direct care provided to a patient with (probable or confirmed COVID-19) disease without using proper personal protective equipment. A significant contact of TB was determined using the standard definition in the 2019 Lesotho guidelines, that includes any household contact of a person who has been bacteriologically confirmed pulmonary TB, proven or suspected drug-resistant TB, person living with HIV or a child below five years [35]. We defined positive pre-screening as the presence of at least one sign or symptom, or a history of close contact with a COVID-19 case. People who pre-screened positive and were 5 years or older, were referred to a nurse, who determined eligibility to enroll in this diagnostic model, explained the testing procedures, and obtained informed consent.

Once the participant was enrolled, the nurse took medical history focusing on COVID-19, TB, and HIV, measured BMI, body temperature, and performed a clinical examination to assess the severity of respiratory symptoms. We defined mild, moderate and severe clinical presentation as follows: 1) mild disease included the presence of COVID-19 signs and symptoms, without hypoxia or dyspnea; 2) moderate disease included the presence of signs and symptoms of pneumonia (fever, cough, dyspnea, tachypnoea) or an oxygen saturation ≤ 94%, with no signs of severe pneumonia; and 3) severe disease included the presence of at least one of the following: altered mental status, oxygen saturation < 94% or signs of central cyanosis, shortness of breath or difficulty breathing, fast breathing (5–9 y ≥ 30 breaths/min; ≥10 y ≥ 20 breaths/min; and ≥18 y ≥ 22 breaths/min), systolic blood pressure <100 mmHg in adults and general danger signs in children. For severely ill participants, the nurse referred them to a physician in the hospital immediately after the clinical examination and patients followed the integrated diagnostic procedures only if the physician judged that these would benefit them. Patients with mild or moderate symptoms at presentation remained in the study and were offered all the clinical procedures. Enrolled participants had a digital posterior-anterior chest Xray done and analyzed using artificial intelligence-powered, computer-aided detection software for TB and COVID-19 (CAD4TB version 6; Delft Imaging Systems, the Netherlands). Participants with a CAD4TB score of 50 were considered as presumptive TB cases and investigated for TB [36].

#### TB, COVID-19, and HIV diagnosis

Diagnosis of SARS-CoV-2 infection was done using RDTs or a polymerase chain reaction test (PCR), in different combinations that varied along the implementation period, adapting to the enrolment of different studies, and the evolving diagnostic recommendations for SARS-CoV-2 in Lesotho. We used the STANDARD Q COVID-19 Antigen test (SD Biosensor, Republic of Korea) with a nasal or a nasopharyngeal swab, and PCR (ABI 7500 Real-Time PCR platform, Applied Biosystem, USA and Xpert Xpress SARS-CoV-2 Cepheid, USA). All participants included in this study had at least one nasopharyngeal SARS-CoV-2 RDT result. Participants who presented with presumptive TB symptoms or a CAD4TB score > 50 were requested to submit sputum for TB diagnostic testing using the Xpert MTB/RIF Ultra (PCR GeneXpert, Cepheid, Sunnyvale, CA, USA). Participants who were requested to submit sputum watched a video describing how to produce quality sputum before they attempted to produce the sample. For HIV diagnosis, we followed the standard of care in Lesotho, that included a first assessment using an HIV risk screening tool to determine HIV testing eligibility. If participants were found to be high risk using the HIV testing screening tool, they were tested following the Lesotho HIV guidelines that used standard blood based RDTs [37]. For the purpose of this study, we defined a COVID-19 case as an individual with a positive result in either RDT (nasopharyngeal or nasal swab) or PCR (nasopharyngeal swab). TB diagnosis was defined as a positive Xpert MTB/RIF Ultra result in sputum, and an HIV diagnosis was defined according to Lesotho standard testing algorithm. After a positive TB and HIV diagnosis, the study staff followed clinical procedures to ensure linkage to care, including counseling and provision of a referral letter to the patient, and coordination among staff of the different departments [38].

#### Follow up after 28 days after testing

To measure the effect of this integrated multi-disease diagnostic service, in terms of linkage to COVID-19 curative services, TB and/or HIV treatment initiation, all enrolled participants that had at least one TB, COVID-19 (RDT or PCR) or HIV positive result, were followed up in a window of time around 28 days after enrolment. A study nurse attempted to contact each participant or their next in kin, through a phone call. In the case of participants diagnosed with COVID-19, we collected information on outcomes defined pragmatically: whether they were “healthy” (had recovered from symptoms, after the episode), “ill” (symptoms persisted but could be managed at home) “hospitalized” (still admitted due to the condition) or “deceased”. For participants that were newly diagnosed with TB or HIV, we asked whether they had started treatment after diagnosis and checked hospital records (TB and HIV registers) at the two study hospitals.

### Ethical considerations

All procedures were carried out in line with the ethical standards laid out in the Declaration of Helsinki [39]. Participants that enrolled in this diagnostic model received information on the clinical procedures in Sesotho and gave written informed consent and those who were illiterate gave consent by thumbprint and a witness signature. Parents or guardians of all participants under 18 years signed an informed consent form, while participants between 7 and 17 years additionally signed an assent form. This project was approved by the Lesotho Ministry of Health Ethics Committee (ID 107–2020) and by the Ethic Committee Switzerland (EthikkommissioNordwest-und Zentralschweiz(EKNZ) AO_2020–00018).

### Data collection and analysis

Clinical data were collected by trained nurses using the Open Data Kit (ODK) software [40]. Information that could not be obtained on the same day, such as Xpert MTB/RIF Ultra and SARS-CoV-2 PCR results, was uploaded to ODK as soon as it became available. Routine data quality checks were done regularly. To compare TB and HIV indicators across hospitals we obtained data from the Lesotho District Health Information System 2(DHIS 2) [41]. Information about social restrictions, COVID-19 policies modification, and COVID-19 national response in Lesotho was compiled using the Lesotho Ministry of Health sources. Descriptive statistics were used to characterize the study sample. We calculated median and interquartile ranges (IQR) to describe continuous variables, and frequencies and percentages for categorical variables. Statistical analyses were performed using Stata (version 16.1, College Station, Tex: StataCorp LP, 2007).

## Results

Of the 179160 adults and children that were pre-screened, a total of 6623 (3.7%) had at least one COVID-19 symptom or history of close contact with a COVID-19 or TB case. Amongst those who pre-screened positive, a total of 4371 (66%) were enrolled in the integrated TB/COVID-19/HIV testing procedures. The remaining 2252 (34%) participants that did not enrolled despite presenting symptoms were: 1) children below five years, 2) unaccompanied children between the age of 7 and 17 years, 3) patients presenting in the weekends, when the service was not available, 4) patients with severe and life-threatening symptoms that required immediate medical attention, or 5) people who refuse to enroll in this service. Table 2 provides an overview of the baseline characteristics of enrolled participants. A total of 2468 (61,7%) were adult women and 369 (8.4%) were children and adolescents between the age of 5 and 17 years. Most of them presented with at least one symptom, most commonly cough, or pronounced tiredness. The median time of symptoms onset was 3 (IQR 2–7) days, and more than 98% presented with mild or moderate symptoms and had an oxygen saturation ≥ 95%. A total of 1768 (44.1%) adults had overweight or obesity (BMI ≥25 Kg/m2), 1058 (27.8%) had one elevated blood pressure measurement (≥140/90 mmHg), and 321 (8.0%) reported current tobacco use. A total of 125 (3.7%) adults and 2 (0.6%) children reported a recent TB contact, while 111 (3.3%) adults and 6 (1.9%) children reported a recent COVID-19 contact. A total of 814 adults (20.3%) and 28 children (7.6%) reported to live with HIV.

**Table 2:** Participants’ baseline characteristics.

Category	5–17.9 years	≥18 years	Total
	(N/%)	(N/%)	(N/%)
	369 (100%)	4002 (100%)	4371 (100%)
Female	241 (65.3%)	2468 (61.7%)	2709 (62.0%)
Male	128 (34.7%)	1534 (38.3%)	1662 (38.0%)
**Symptoms at presentation** N = 4193			
At least one symptom	322 (92.5%)	3537 (91.8%)	3859 (91.8%)
No symptoms	26 (7.5%)	318 (8.2%)	344 (8.2%)
Days onset of any symptoms (Median time, IQR)	3 (2–4)	4 (2–7)	3 (2–7)
Fever	0 (0.0%)	10 (0.3%)	10 (0.3%)
Cough	231 (68.1%)	2289 (61.6%)	2520 (62.1%)
Pronounced tiredness	49 (14.5%)	924 (25.4%)	973 (24.4%)
Shortness of breath	17 (5.1%)	381 (10.7%)	398 (10.2%)
Chest pain	11 (3.2%)	268 (7.0%)	279 (6.6%)
Fatigue	28 (8.0%)	721 (18.7%)	749 (17.8%)
Sore throat	62 (18.3%)	604 (16.8%)	666 (16.9%)
Muscle pain	3 (0.9%)	311 (8.1%)	314 (7.5%)
Diarrhoea	18 (5.3%)	169 (4.8%)	187 (4.8%)
Loss of sense or smell	14 (4.2%)	195 (5.5%)	209 (5.4%)
Runny nose	11 (3.2%)	98 (2.5%)	109 (2.6%)
Headache	27 (7.8%)	349 (9.1%)	376 (8.9%)
Skin rash	2 (0.6%)	8 (0.2%)	10 (0.2%)
Weight loss	8 (2.4%)	171 (4.8%)	179 (4.6%)
Night sweats	6 (1.8%)	178 (5.0%)	184 (4.7%)
Vomiting	9 (2.6%)	54 (1.4%)	63 (1.5%)
**Clinical severity at presentation**^**a**^ N = 4157			
Signs of clinical severity	18 (5.1%)	62 (1.6%)	80 (1.9%)
No signs of clinical severity	334 (94.9%)	3743 (98.4%)	4077 (98.1%)
**Pregnancy (reported)** N = 2450			
Yes	4 (3.6%)	88 (3.8%)	92 (3.8%)
No	106 (96.4%)	2252 (96.2%)	2358 (96.2%)
**BMI (adults)** N = 3950			
<18.5 Kg/m^2^	-	411 (10.4%)	411 (10.4%)
18.5–24.9 Kg/m^2^	-	1771 (44.8%)	1771 (44.8%)
≥25–29.9 Kg/m^2^	-	970 (24.6%)	970 (24.6%)
≥30 Kg/m^2^	-	798 (20.2%)	798 (20.2%)
**Blood pressure (systolic/diastolic, adults)** N = 3805			
<140/90 mmHg	-	2747 (72.2%)	2747 (72.2%)
≥140/90 mmHg	-	1058 (27.8%)	1058 (27.8%)
**Oxygen saturation (capillary)** N = 4157			
<85%	2 (0.6%)	41 (1.1%)	43 (1.0%)
85 - <90%	0 (0.0%)	54 (1.4%)	54 (1.3%)
90 - <95%	82 (23.3%)	898 (23.6%)	980 (23.6%)
≥95%	268 (76.1%)	2812 (73.9%)	3080 (74.1%)
**BCG Vaccine (reported, visible scar)** N = 3741			
Yes	305 (93.6%)	3103 (90.9%)	3408 (91.1%)
**Recent TB contact (reported)** N = 3741			
Yes	2 (0.6%)	125 (3.7%)	127 (3.4%)
**Recent COVID-19 contact (reported)** n = 3691			
Yes	6 (1.9%)	111 (3.3%)	117 (3.2%)
**Past episode TB (reported)** n = 4051			
Yes	1 (0.3%)	341 (9.2%)	342 (8.5%)
**Comorbidities (reported**) n = 645			
Diabetes	1 (0.3%)	119 (3.5%)	120 (3.3%)
Hypertension	0 (0.0%)	499 (14.8%)	499 (13.5%)
Heart disease	0 (0.0%)	24 (0.7%)	24 (0.7%)
Cancer	0 (0.0%)	2 (0.1%)	2 (0.1%)
**HIV status** N = 4371			
Positive (reported)	28 (7.6%)	814 (20.3%)	842 (19.3%)

^a^ Defined as presence of at least one of the following: altered mental status, respiratory rate ≥ 22 cycles per minute, shortness of breath or difficulty breathing, oxygen saturation < 94% and systolic blood pressure <100 mmHg for adults and signs of pneumonia and central cyanosis, oxygen saturation < 94%, severe respiratory disease, general danger signs, tachypnea in children (5–9 y ≥ 30 breaths/min, ≥10y ≥ 20 breaths/min)

### Integrated COVID-19/TB and HIV screening and testing model

A summary of the diagnostic results is available in Table 3. In terms of SARS-CoV-2 testing, a total of 4355, 2421 and, 2664 nasopharyngeal RDT, nasal RDT, and nasopharyngeal PCR tests were done. With regards to TB investigations, a total of 4371 chest x-rays were performed, and overall, 884 (29.3%) participants had a CAD4TB score > 50. For the 2419 participants who were eligible for sputum collection, 1282 samples were collected and tested with Xpert MTB/RIF Ultra. Most common reasons for not collecting sputum included patient refusing (377; 15,8%) or unable to produce a sample (263; 10.8%). A total of 106 people were tested for HIV. Overall, a total of 458 new diagnoses were made, of which 383, 51 and 24 were COVID-19, TB, and HIV cases, respectively (Table 3). Newly found cases were mainly diagnosed among adults. Only 16 (4.3%) of the COVID-19 cases and one (1%) of the TB cases were diagnosed in children and adolescent participants, and this model did not find any new HIV diagnosis amongst them.

**Table 3:** COVID-19, TB, and HIV diagnosis, disaggregated by age groups.

	5–17 years (n/%)	≥18 years (n/%)	Total (n/%)
**Enrolled in integrated diagnostic model**	369 (100%)	4002 (100%)	4371 (100%)
**SARS-CoV-2 diagnostic tests done:**			
SARS-CoV-2 NP PCR (n = 2664)	4/227 (1.8%)	178/2437 (7.3%)	182 (6.8%)
SARS-CoV-2 NP rapid test (n = 4355)	15/369 (4.1%)	298/3986 (7.5%)	313 (7.2%)
SARS-CoV-2 Nasal rapid test (n = 2421)	4/213 (1.9%)	126/2208 (5.7%)	130 (5.4%)
**TB diagnostic tests done**			
Number of Xray done	369	4002	4,371
CAD4TB score ≤ 50	288 (78.0%)	2092 (52.2%)	2380 (54.4%)
CAD4TB score > 50	15 (4.1%)	869 (21.7%)	884 (20.2%)
Missing/ not evaluated	66 (17.9%)	1041 (26.0%)	1107 (25.3%)
N of participants eligible to collect sputumᶛ	168	2251	2419
Xpert done/sputum collected^Ɣ^	78	1204	1282
Xpert positive DSTB (no RR)	0 (0.0%)	41 (3.4%)	41 (3.2%)
Xpert positive RR	1 (1.3%)	7 (0.6%)	8 (0.6%)
Xpert positive, RR. indeterminate	0 (0.0%)	2 (0.2%)	2 (0.2%)
Xpert trace*	0 (0.0%)	3 (0.2%)	3 (0.2%)
Xpert negative	77 (98.7%)	1146 (95.2%)	1223 (95.4%)
Xpert invalid	0 (0.0%)	5 (0.4%)	5 (0.4%)
**HIV Diagnostics**			
HIV test done	6 (100.0%)	100 (100.0%)	106 (100.0%)
HIV result positive	0 (0.0%)	24 (24.0%)	24 (22.6%)
HIV results negative	6 (100.0%)	76 (76.0%)	82 (77.4%)
**New cases found** ^**μ**^			
COVID-19	16 (4.3%)	367 (9.2%)	383 (8.8%)
TB	1 (1%)	50 (3.8%)	51 (3.6%)
HIV	0 (0.0%)	24 (24.0%)	24 (22.6%)
**Total**	17 (4.6%)	441 (11%)	458 (10.4%)
**28 days follow up**			
Number of people reached after 4 weeks^$^	11 (64.7%)	310 (83.8%)	321 (82.9%)
Recovered health^ß^	10 (90.9%)	294 (94.8%)	304 (94.7%)
Deceased	1 (9.1%)	11 (3.5%)	12 (3.7%)
**Linkages to TB and HIV treatment**			
Started ART	0 (0%)	18/24 (75.0%)	18/24 (75.0%)
Started TB treatmentᶾ	1/1 (100%)	45/50 (90.0%)	46/51 (90.2%)

* Xpert “trace” results prompted further clinical evaluation

ᶛ Symptoms or CAD4TB >50

^Ɣ^ including samples without a result

^μ^ Including 8 adults with known HIV+ status that tested positive for TB and 12 adults and 1 child who tested positive for TB whose HIV status was unknown.

^$^Total number of people they reached, of those who the study nurses tried to contact.

^ß^ Participants reported that they had recovered from the episode that had prompted the hospital visit

ᶾ People with drug-resistant TB started TB treatment at a DRTB referral hospital

With regards to the clinical outcomes after four weeks of diagnosis, of the 458 people who were diagnosed with either COVID-19, TB or HIV, a total of 321 (82.9%) participants, or their relatives could be reached. Of those, 304 (94.7%) reported that they were healthy, whereas 5 (1.3%) reported to be ill or hospitalized (four people diagnosed with COVID-19 and one person who was HIV positive). We recorded 12 (3.7%) participants who died in this period, however we did not record the possible causes of death. Of the individuals that were newly diagnosed with HIV or TB, a total of 18/24 (75.0%) and 46/51 (90.2%) had started ART and TB treatment respectively, during this period (Table 3).

**Fig 2:**
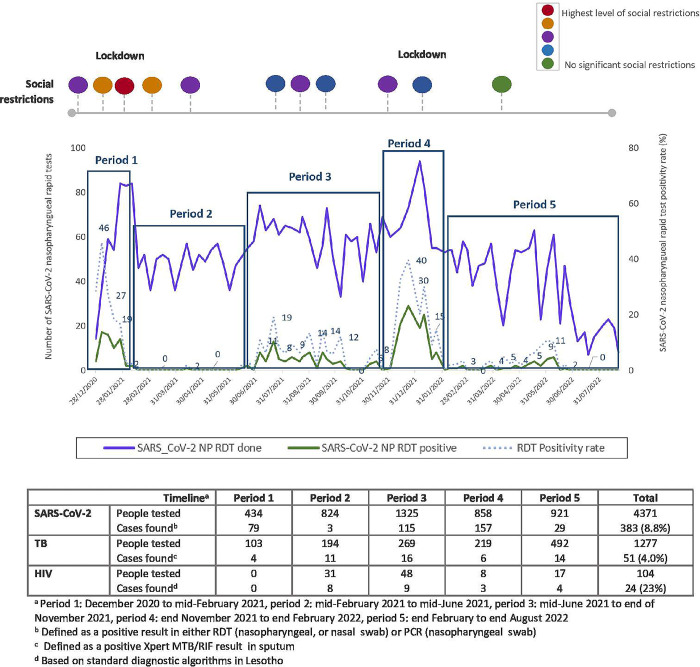
COVID-19, TB and HIV cases diagnosed along different time periods MistraL project.

Overall, one positive rapid antigen test for SARS-CoV-2 was found per 11 participants enrolled, one Xpert-positive TB case was diagnosed per 85 people enrolled, and 1 new HIV diagnosis was done per 182 people enrolled. However, the yield of this integrated testing model in terms of additional HIV, TB, and COVID-19 diagnosis across periods of different COVID-19 incidence is displayed in Fig 2. Periods 1 and 4 (end of 2020 to mid-February 2021, and end of November 2021 to end of February 2022), are characterized by high SARS-COV-2 RDT positivity rates (20–40%), during very short periods of time (distinct COVID-19 waves). Periods 3 and 5 (mid-June to end of November 2021, and end of February to end of August 2022), are marked by lower SARS-COV-2 RDT positivity rates across longer periods of time, indicating ongoing, low level of community transmission. During period 2 (mid-February 2021 to Mid-June 2021), there were almost no COVID-19 cases diagnosed at our sites. During periods of high COVID-19 incident cases (1 and 4), when study staff had to cope with a larger number of enrolled participants, the number of TB and HIV additional cases found was irregular, whereas the periods of lower COVID-19 incident cases allowed for a higher number of new TB and HIV diagnosis.

S1 Fig offers a contextualization of the performance for HIV and TB new notifications, as well as the ART initiations, reported in six hospitals Lesotho during the period of October 2020 to March 2022, using the Lesotho DHIS-2 reporting system. The two study sites (Seboche Mission Hospital and Mokhotlong Hospital) where integrated diagnosis occurred are plotted in context with Paray, St. James, Ntseke, and Tellebong hospitals, where COVID-19 testing was conducted, but not integrated with TB and HIV services. These hospitals are similar to the study sites. Each also gives services in the highlands to an estimated population of 14,000 to 25,000 people living in mostly rural communities in the districts of Thaba Tseka (Paray and St James Hospitals), Mohale’s Hoek (Ntseke Hospital) and Qacha’s Nek (Tebellong Hospital). They are also very similar in terms of inpatient capacity, between 70–120 beds [42]. This data shows that all hospitals maintained HIV and TB services similarly during this period.

## Discussion

This observational study aimed to describe and assess the impact of an integrated COVID-19, TB and HIV screening and testing service model in two hospitals in rural Lesotho, during the period between December 2020 and August 2022. Our setting was characterized by an overall low incidence of COVID-19 cases, a volatile context, and social restrictions in continuous revision. Nonetheless, this service operated during two distinct COVID-19 waves and two other three periods when COVID-19 incident cases were continuously low. This pragmatic diagnostic service model provided integrated diagnostic services to 4,371 adults and children, who most frequently presented with non-severe symptoms, and previous contact with a TB or COVID-19 case was infrequently reported. The overall diagnostic yield, including COVID-19, TB, and HIV, was 458 new cases, most frequently found in adults; only 17 (3.7%) of them happened in children and adolescents. Lastly, this model introduced changes that potentially improved infection control and reduced nosocomial transmission for both for TB and COVID-19 at the study sites.

It is almost certain that in this study we underestimate the number of COVID-19 and TB cases diagnosed. In the case of COVID-19 cases, a PCR test was used as the gold standard for diagnosis during most part of 2021. Later on, diagnosis was based on the result of the SARS-CoV-2 STANDARD Q COVID-19 Ag test, that has shown relatively low diagnostic performance in our setting [43]. Anecdotically, some of the 2252 (34%) eligible adults and children that presented with symptoms but did not enroll in this service model despite being eligible, were still tested for COVID-19 by the study staff, especially in periods were hospital health staff in other departments was scarce, however, these individuals are not included in this analysis. With regards to the definition of TB case in this study, we report only positive Xpert MTB/RIF Ultra results in the sputum of participants, from whom we obtained a sample. In our case, sputum samples could not be obtained for one third of the participants, mainly due to refusals or inability to produce them. Additionally, for this analysis, we did not consider a clinical TB diagnosis made by clinicians once a symptomatic patient was referred to further evaluation by hospital staff. Similarly, we may have underestimated the rate of linkage to TB and/or HIV care for newly diagnosed individuals, who after the diagnosis could have seeked services elsewhere, especially in surrounding primary health facilities.

To interpret these findings, it is also important to consider broader contextual factors that played a role. One of them is related to the variation of circulating SARS-CoV-2 virus (Omicron variant) and the lower diagnostic sensitivity observed using RDTs [44–46]. In our setting, this could have led to a lower detection of cases in symptomatic people seeking services. A second factor could be related to the fluctuating access to hospital services. It is probable that the changes imposed by the social restrictions, the availability of service providers (themselves ill, in quarantine, or isolated due to a recent contact), or the fear to acquire a COVID-19 infection in the hospital, were important deterrents for symptomatic people to seek hospital services.

Of the 321 (82.9%) participants that were successfully reached after four weeks of the diagnosis, 304 (94.7%), 18/24 (75%), and 46/53 (86.8%) were in good health, had started ART and TB treatment respectively during this period. We recorded 12 (3.6%) participants deaths, however, around one in six participants with a diagnosis could not be contacted in the follow up period, therefore these results need to be interpreted with caution. Using the total number of 4371 enrolled participants as a benchmark, we calculate that we found one SARS-CoV-2 rapid antigen positive per 11 people screened, one Xpert MTB/RIF Ultra positive test per 85 people screened, and 1 HIV new diagnosis was found per 182 people screened.

Surprisingly, we found no clear evidence to suggest that this resource-intense screening and testing model had a significantly different impact in sustaining access to TB and HIV diagnostic and treatment services, compared to similar hospitals in Lesotho, where this diagnostic model was not in place, when referring to the Lesotho TB and HIV DHIS-2 reports. During the analyzed period of October 2020 to March 2022, the number of HIV new diagnosis, ART initiations, and TB case notifications in the six hospitals seem quite similar. Additionally, the results from our study suggest that this diagnostic model had little impact on pediatric and adolescent COVID-19, TB, and HIV case finding in participants who were between the age of 5 and 18 years, although the numbers are small. On the other hand, the overall TB and HIV linkage to care rates within four weeks of diagnosis were higher than reported in similar experiences [47–50], however when comparing to global targets, this model fell short in ART initiation rates (75% vs 95%), and met the TB treatment initiation target (90% vs. 90%) [51, 52].

As the COVID-19 pandemic surged, and lockdowns led to the cancellation of essential health services, global health actors advocated to adapt services that integrated TB, HIV and COVID-19 activities in sub-Saharan Africa [29, 42–44]. This established an emerging body of evidence that evaluates the experiences and successes in the integration of services. A study in Uganda, using qualitative methods [33], reported that facilitators to integrate COVID-19 and TB services included availability of focal health workers who were solely responsible for this task, and availability of standard procedures and data collection tools. In this setting, inconsistent supply of protective gear and fear of contracting COVID-19 decreased the efficiency of the service. A study in Ethiopia reported a sharp reduction of TB service indicators in Addis Ababa, partially mitigated with the use of digital health technology to screen for TB, however, their results were not conclusive, due to lack of patient-level data [32]. Another similar experience in Niger and Guinea [53], found that out of the 863 individuals enrolled, 61 (7%) tested positive for COVID-19 and 43 (4.9%) were diagnosed with TB. These results are comparable to our COVID-19 notifications in Lesotho (383, 8.8%), however, the investigators in Niger and Guinea found a higher number of TB cases than in our setting (35 (4.6%) in Guinea, 8 (7.6%) in Niger, and 51 (1.1%) in Lesotho). Reports of pragmatic integrated experiences aiming to sustain HIV testing and linkage to treatment amid the COVID-19 epidemic across sub-Saharan Africa are very scarce. A systematic review evaluating the impact of the COVID-19 pandemic on accessing HIV services in South Africa, and an evaluation of performance of services in primary health clinics [13, 54] revealed that there were significant decreases in HIV testing, positive HIV tests, and ART initiation at public health facilities. However, the private facilities had maintained their activity similarly to pre-pandemic levels, confirming a disparity in health access between the private and public sectors. In our service, the routine use of the HIV testing screening tool targeted individuals who were most at risk of a recent infection and yet, one in five people tested positive for HIV. However, it is important to note that in our setting performing HIV testing depended greatly on the availability of time that the study nurses had, while being burdened with other tasks. In periods of sharp COVID-19-related overload, created by an increase of influx of individuals looking to be tested, participants who were also eligible for HIV testing were referred to the hospital HIV testing services. This was a result of an effort to maintain the function of the COVID-19 diagnostic services and the quality of the HIV testing service. This typically happened in a different hospital department and could have decreased the efficiency to find new HIV cases. Similarly to other published studies in sub-Saharan Africa and other regions of the world [32–34, 53, 55–57], our integrated screening and diagnostic model proved to be a resource intensive service, that had to adapt to contextual factors continuously. To ensure availability and continuity of the service, we made significant investments in the deployment, protection, training, and support of health staff.

As the emergency COVID-19 phase came to an end, there is a need to draw conclusions about whether similar care models would be appropriate, affordable, and effective in future similar situations. This can support evidence-based decisions for countries’ emergency preparedness plans. For example, our care model was designed to only sustain services for other prevalent infectious diseases (TB and HIV). Despite the routine collection of BMI and BP, these values were only used to evaluate the clinical severity and risk of the respiratory condition at presentation. This model of care did not integrate specific medical services for undiagnosed or untreated cardiovascular risks, also prevalent in this setting. However, integrating an even more inclusive care model could have failed to meet the needs for SARS-CoV-2 testing, especially during the periods with highest work burden or severe shortages of clinical staff. Furthermore, we hypothesize, that access to available health services was very dependent on the social measures applied in each period, especially when movements were restricted, or people with respiratory symptoms experienced fear around nosocomial transmission of SARS-CoV-2, or the impact of imposed quarantines. The question of how best and for how long to integrate medical services in acute emergencies, with volatile and fast-changing contexts, remains open. Future studies should evaluate how to integrate more robust approaches to the evaluation of service provision in such situations, particularly focusing on impact on morbidity, mortality, quality of life, and effects in health systems.

Our study had various limitations. A major weakness is that we report on a hospital-based screening and testing model, that served the population who voluntarily reached and enrolled in the service. Therefore, our results refer to a selected number of individuals enrolled in each period of time. Similarly, data systems were not designed to report on individuals who seeked care at these hospitals several times during the study period. By the end of 2021, SARS-CoV-2 ag-RDTs became available at community level, through local retailers and primary health clinics. As capacity for rapid testing for COVID-19 expanded, the population living in these districts could choose where and how to receive this service. Secondly, complementing our study with qualitative approaches could have increased the efficiency, and acceptability of the integrated diagnostic service, and explored the level and consequences of stigma related to COVID-19 in our setting. However, these aspects were explored from the perspective of the community in the two districts and will be reported in other studies. Thirdly, detailed hospital-based data on COVID-19, TB, and HIV, such as number of SARS-CoV-2 tests by period, number of TB cases diagnosed using Xpert tests in sputum, or age-disaggregated HIV tests could not be obtained from surrounding hospitals in other districts. This limits possible comparisons of performance during the epidemic period, and the use of DHIS2 data rather offers information about the context in which these hospitals operated. Fourthly, we report non-negligible death rate, however, we do not have enough information to distinguish between non-related to presenting illness, such as trauma deaths, (e.g., road traffic injury, violence, suicide) and non-traumatic deaths that could be attributable to the infectious causes. Lastly, an economic analysis of this model could shed light on important questions related to health system-related costs and patients-related costs. Despite these limitations, this is the first study evaluating the effect of an integrated, COVID-19/TB and HIV diagnostic model in sub-Saharan Africa, covering a period of 22 months and reporting on disaggregated characteristics and outcomes in adults and children above five years.

## Conclusion

This screening and diagnostic model successfully maintained same-day, integrated COVID-19, TB, and HIV testing services, despite frequent disruptions caused by the surge of COVID-19 waves, healthcare seeking patterns, and the volatile context (social measures, travel restrictions, population lockdowns). There were positive effects in avoiding diagnostic delays and ensuring linkage to services, however, diagnostic yields for adults and children were low. To inform future preparedness plans, research will need to identify essential health interventions and how to optimize them along each phase of the emergency response.

## Supporting information

S1 Fig Trends for HIV and TB services at 6 hospitals in Lesotho in the period between October 2020 and March 2022: HIV tests, ART initiations, and TB notifications. (TIFF)

## References

[1] Kendzerska T. et al., “The Effects of the Health System Response to the COVID-19 Pandemic on Chronic Disease Management: A Narrative Review,” *Risk Manag Healthc Policy*, vol. 14, pp. 575–584, 2021, doi: 10.2147/RMHP.S293471 33623448 PMC7894869

[2] Moynihan R. et al., “Impact of COVID-19 pandemic on utilisation of healthcare services: a systematic review,” *BMJ Open*, vol. 11, no. 3, p. e045343, Mar. 2021, doi: 10.1136/bmjopen-2020-045343 33727273 PMC7969768

[3] Arsenault C. et al., “COVID-19 and resilience of healthcare systems in ten countries,” *Nat Med*, vol. 28, no. 6, pp. 1314–1324, Jun. 2022, doi: 10.1038/s41591-022-01750-1 35288697 PMC9205770

[4] “Third round of the global pulse survey on continuity of essential health services during the COVID-19 pandemic: November—December 2021: interim report, 7 February 2022—World | ReliefWeb.” https://reliefweb.int/report/world/third-round-global-pulse-survey-continuity-essential-health-services-during-covid-19 (accessed Dec. 02, 2022).

[5] World Health Organization, “Maintaining essential health services: operational guidance for the COVID-19 context,” *World Health Organization*, no. June, p. 55, 2020.

[6] PATH, “Essential health services during and after COVID-19: A sprint analysis of disruptions and responses across six countries,” no. December, p. 26, 2020.

[7] “Global Tuberculosis Report 2022.” https://www.who.int/teams/global-tuberculosis-programme/tb-reports/global-tuberculosis-report-2022 (accessed Dec. 01, 2022).

[8] “HIV and COVID-19.” https://www.who.int/teams/global-hiv-hepatitis-and-stis-programmes/covid-19 (accessed Dec. 01, 2022).

[9] “Essential health services during and after COVID-19: A sprint analysis of disruptions and responses across six countries.” https://www.path.org/resources/essential-health-services-during-and-after-covid-19-sprint-analysis-disruptions-and-responses-across-six-countries/ (accessed Apr. 01, 2023).

[10] Nachega J., Seydi M., and Zumla A., “The Late Arrival of Coronavirus Disease 2019 (COVID-19) in Africa: Mitigating Pan-continental Spread,” *Clin Infect Dis*, vol. 71, no. 15, pp. 875–878, Jul. 2020, doi: 10.1093/cid/ciaa353 32227121 PMC7184327

[11] Holtzman C. W. et al., “PEPFAR’s Role in Protecting and Leveraging HIV Services in the COVID-19 Response in Africa,” *Curr HIV/AIDS Rep*, vol. 19, no. 1, pp. 26–36, 2022, doi: 10.1007/s11904-021-00587-6 34982406 PMC8724594

[12] Tessema G. A. et al., “The COVID-19 pandemic and healthcare systems in Africa: A scoping review of preparedness, impact and response,” *BMJ Global Health*, vol. 6, no. 12, pp. 1–14, 2021, doi: 10.1136/bmjgh-2021-007179 34853031 PMC8637314

[13] Dorward J. et al., “The impact of the COVID-19 lockdown on HIV care in 65 South African primary care clinics: an interrupted time series analysis,” *The Lancet HIV*, vol. 8, no. 3, pp. e158–e165, Mar. 2021, doi: 10.1016/S2352-3018(20)30359-3 33549166 PMC8011055

[14] “Population, total—Lesotho | Data.” https://data.worldbank.org/indicator/SP.POP.TOTL?locations=LS (accessed Dec. 14, 2022).

[15] “TB profile.” https://worldhealthorg.shinyapps.io/tb_profiles/?_inputs_&entity_type=%22country%22&lan=%22EN%22&iso2=%22LS%22 (accessed Nov. 25, 2022).

[16] Schwitters A. et al., “High HIV prevalence and associated factors in Lesotho: Results from a population-based survey,” *PLOS ONE*, vol. 17, no. 7, p. e0271431, Jul. 2022, doi: 10.1371/journal.pone.0271431 35901094 PMC9333200

[17] “Lesotho.” https://www.unaids.org/en/regionscountries/countries/lesotho (accessed Dec. 11, 2022).

[18] “COVID-19 Preparedness in Lesotho,” THET. https://www.thet.org/case-studies/covid-19-preparedness-in-lesotho/ (accessed Nov. 26, 2022).

[19] “Family planning uptake drops in Lesotho due to COVID-19 restrictions, analysis shows—Lesotho | ReliefWeb.” https://reliefweb.int/report/lesotho/family-planning-uptake-drops-lesotho-due-covid-19-restrictions-analysis-shows (accessed Nov. 26, 2022).

[20] Sanders J. E. et al., “National hospital readiness for COVID-19 in Lesotho: Evidence for oxygen ecosystem strengthening.” medRxiv, p. 2021.04.27.21256199, Apr. 30, 2021. doi: 10.5588/pha.21.0067 34956845 PMC8680183

[21] Nyangu I. and Ramathebane M., “Perceptions of Healthcare Professionals on COVID-19 in Lesotho: A Cross-Sectional Survey,” *Journal of Biosciences and Medicines*, vol. 10, no. 2, Art. no. 2, Jan. 2022, doi: 10.4236/jbm.2022.102005

[22] “Lesotho-Humanitarian-SitRep-June-2021.pdf.” Accessed: Dec. 11, 2022. [Online]. Available: https://www.unicef.org/media/104171/file/Lesotho-Humanitarian-SitRep-June-2021.pdf

[23] “Lesotho: WHO Coronavirus Disease (COVID-19) Dashboard With Vaccination Data.” https://covid19.who.int (accessed Nov. 26, 2022).

[24] A. P. Release, “Lesotho Moves to Green Code Colour Stage.” https://www.zawya.com/en/press-release/africa-press-releases/lesotho-moves-to-green-code-colour-stage-g26m14mn (accessed Nov. 26, 2022).

[25] “LESOTHO MOVES TO BLUE COLOUR CODING STAGE–Government Of Lesotho.” https://www.gov.ls/lesotho-moves-to-blue-colour/ (accessed Nov. 26, 2022).

[26] “Lesotho_2020.08.24_Regulation_Public-Health-COVID-19-Risk-Determination-and-Mitigation-Measures-No.-3-Regulations-2020_EN_.pdf.” Accessed: Nov. 26, 2022. [Online]. Available: https://covidlawlab.org/wp-content/uploads/2022/01/Lesotho_2020.08.24_Regulation_Public-Health-COVID-19-Risk-Determination-and-Mitigation-Measures-No.-3-Regulations-2020_EN_.pdf

[27] “Public-Health-Covid-19-risk-Determination-and-Mitigation-Measure-No.5-Amendment-Regulations-2022.pdf.” Accessed: Nov. 26, 2022. [Online]. Available: https://kleingeldmayet.co.ls/wp-content/uploads/2022/01/Public-Health-Covid-19-risk-Determination-and-Mitigation-Measure-No.5-Amendment-Regulations-2022.pdf

[28] NNN, “Lesotho moves to green code color stage,” NNN, Aug. 24, 2022. https://nnn.ng/lesotho-moves-to-green-code-color-stage/ (accessed Nov. 26, 2022).

[29] “MistraL: Mitigation Strategies for Communities With COVID-19 Transmission in Lesotho Using Artificial Intelligence on Chest X-rays and Novel Rapid Diagnostic Tests–BRCCH.” https://brc.ch/research/mistral/ (accessed Nov. 26, 2022).

[30] Ruhwald M., Hannay E., Sarin S., Kao K., Sen R., and Chadha S., “Considerations for simultaneous testing of COVID-19 and tuberculosis in high-burden countries,” *The Lancet Global Health*, vol. 10, no. 4, pp. e465–e466, Apr. 2022, doi: 10.1016/S2214-109X(22)00002-X 35122718 PMC8809899

[31] MacLean E. L., Villa-Castillo L., Ruhwald M., Ugarte-Gil C., and Pai M., “Integrated testing for TB and COVID-19,” *Med (N Y)*, vol. 3, no. 3, pp. 162–166, Mar. 2022, doi: 10.1016/j.medj.2022.02.002 35169763 PMC8831149

[32] Arega B. et al., “Impact of COVID-19 pandemic on TB prevention and care in Addis Ababa, Ethiopia: a retrospective database study,” *BMJ Open*, vol. 12, no. 2, p. e053290, Feb. 2022, doi: 10.1136/bmjopen-2021-053290 35135769 PMC8829833

[33] Semitala F. C. et al., “Integration of COVID-19 and TB screening in Kampala, Uganda—Healthcare provider perspectives,” *Res Sq*, p. rs.3.rs-1448831, Jun. 2022, doi: 10.21203/rs.3.rs-1448831/v1 36650596 PMC9844180

[34] Njau A., Kimeu J., Gohil J., and Nganga D., “Informing healthcare operations with integrated pathology, clinical, and epidemiology data: Lessons from a single institution in Kenya during COVID-19 waves,” *Front Med (Lausanne)*, vol. 9, p. 969640, 2022, doi: 10.3389/fmed.2022.969640 36148453 PMC9485835

[35] “TB Guidelines 2019 –Ministry of Health.” http://health.gov.ls/download/tb-guidelines-2019/ (accessed Apr. 17, 2023).

[36] Qin Z. Z. et al., “Tuberculosis detection from chest x-rays for triaging in a high tuberculosis-burden setting: an evaluation of five artificial intelligence algorithms,” *The Lancet Digital Health*, vol. 3, no. 9, pp. e543–e554, Sep. 2021, doi: 10.1016/S2589-7500(21)00116-3 34446265

[37] “Linkages across the Continuum of HIV Services for Key Populations Affected by HIV (LINKAGES): Country Resources,” FHI 360. https://www.fhi360.org/resource/linkages-across-continuum-hiv-services-key-populations-affected-hiv-linkages-country (accessed Dec. 02, 2022).

[38] “Lesotho-COP22-SDS.pdf.” Accessed: Apr. 02, 2023. [Online]. Available: https://www.state.gov/wp-content/uploads/2022/09/Lesotho-COP22-SDS.pdf

[39] “WMA—The World Medical Association-WMA Declaration of Helsinki–Ethical Principles for Medical Research Involving Human Subjects.” https://www.wma.net/policies-post/wma-declaration-of-helsinki-ethical-principles-for-medical-research-involving-human-subjects/ (accessed Nov. 25, 2022).

[40] “Open Data Kit,” *Open Data Kit*, Mar. 01, 2018. https://opendatakit.org/ (accessed Dec. 01, 2022).

[41] “Improving DHIS2 Integration for Lesotho’s Health Information Ecosystem—Implementation—Implémentation / Interoperability,” *DHIS2 Community*, May 31, 2022. https://community.dhis2.org/t/improving-dhis2-integration-for-lesothos-health-information-ecosystem/47463 (accessed Nov. 25, 2022).

[42] “PER-of-Health-in-Lesotho-(2017).pdf.” Accessed: Apr. 17, 2023. [Online]. Available: https://www.unicef.org/esa/sites/unicef.org.esa/files/2019-04/PER-of-Health-in-Lesotho-%282017%29.pdf

[43] Labhardt N. D. et al., “Head-to-head comparison of nasal and nasopharyngeal sampling using SARS-CoV-2 rapid antigen testing in Lesotho.” medRxiv, p. 2021.12.29.21268505, Jan. 01, 2022. doi: 10.1101/2021.12.29.21268505 PMC9980827 36862684

[44] “Use of Rapid Antigen Tests during the Omicron Wave,” *Ontario COVID-19 Science Advisory Table*. https://covid19-sciencetable.ca/sciencebrief/use-of-rapid-antigen-tests-during-the-omicron-wave/ (accessed Dec. 13, 2022).

[45] Bekliz M. et al., “Analytical Sensitivity of Eight Different SARS-CoV-2 Antigen-Detecting Rapid Tests for Omicron-BA.1 Variant,” *Microbiology Spectrum*, vol. 10, no. 4, pp. e00853–22, Aug. 2022, doi: 10.1128/spectrum.00853-22 35938792 PMC9430749

[46] Labhardt N. D. et al., “Head-to-head comparison of nasal and nasopharyngeal sampling using SARS-CoV-2 rapid antigen testing in Lesotho,” *PLoS One*, vol. 18, no. 3, p. e0278653, 2023, doi: 10.1371/journal.pone.0278653 36862684 PMC9980827

[47] Chawla K. S. et al., “Policy to practice: impact of GeneXpert MTB/RIF implementation on the TB spectrum of care in Lilongwe, Malawi,” *Transactions of The Royal Society of Tropical Medicine and Hygiene*, vol. 110, no. 5, pp. 305–311, May 2016, doi: 10.1093/trstmh/trw030 27198215 PMC4914880

[48] Shapiro A. E. et al., “Completion of the tuberculosis care cascade in a community‐based HIV linkage‐to‐care study in South Africa and Uganda,” *J Int AIDS Soc*, vol. 21, no. 1, p. e25065, Jan. 2018, doi: 10.1002/jia2.25065 29381257 PMC5810338

[49] Katende B., Esterhuizen T. M., Dippenaar A., and Warren R. M., “Rifampicin Resistant Tuberculosis in Lesotho: Diagnosis, Treatment Initiation and Outcomes,” *Sci Rep*, vol. 10, no. 1, Art. no. 1, Feb. 2020, doi: 10.1038/s41598-020-58690-4 32024860 PMC7002499

[50] Mabote N., Mamo M., Nsakala B., Lanje S., Mwanawabene N. R., and Katende B., “Linkage to care and treatment outcomes for patients diagnosed with drug-susceptible tuberculosis using Xpert MTB/RIF assay in Thaba-Tseka district in Lesotho,” *IJID Regions*, vol. 5, pp. 33–38, Dec. 2022, doi: 10.1016/j.ijregi.2022.08.010 36158597 PMC9493056

[51] “LESOTHO TB Dashboard.” https://www.stoptb.org/static_pages/LSO_Dashboard.html (accessed Nov. 26, 2022).

[52] “Lesotho.” https://www.unaids.org/en/regionscountries/countries/lesotho (accessed Nov. 26, 2022).

[53] Magassouba A. S. et al., “Evaluating the Effectiveness of a Novel Systematic Screening Approach for Tuberculosis among Individuals Suspected or Recovered from COVID-19: Experiences from Niger and Guinea,” *Tropical Medicine and Infectious Disease*, vol. 7, no. 9, Art. no. 9, Sep. 2022, doi: 10.3390/tropicalmed7090228 36136639 PMC9504611

[54] Jardim C. G. R., Zamani R., and Akrami M., “Evaluating the Impact of the COVID-19 Pandemic on Accessing HIV Services in South Africa: A Systematic Review,” *Int J Environ Res Public Health*, vol. 19, no. 19, p. 11899, Sep. 2022, doi: 10.3390/ijerph191911899 36231201 PMC9565529

[55] Khobragade R. N., Murthy N., Aloysius S., Surendran D., Rakesh P. S., and Balakrishnan S., “Experience of integrated screening and testing for TB and COVID19 from Kerala, India,” *Public Health Pract (Oxf)*, vol. 2, p. 100198, Oct. 2021, doi: 10.1016/j.puhip.2021.100198 34632434 PMC8486580

[56] Malik A. A. et al., “Integrated Tuberculosis and COVID-19 Activities in Karachi and Tuberculosis Case Notifications,” *Trop Med Infect Dis*, vol. 7, no. 1, p. 12, Jan. 2022, doi: 10.3390/tropicalmed7010012 35051128 PMC8778721

[57] Muñiz-Salazar R. et al., “Impact of COVID-19 on tuberculosis detection and treatment in Baja California, México,” *Front Public Health*, vol. 10, p. 921596, Jul. 2022, doi: 10.3389/fpubh.2022.921596 35942259 PMC9356343

