# Supplementary figures and images for: Impact of a multi-disease integrated screening and diagnostic model for COVID-19, TB, and HIV in Lesotho
Source: PLOS Glob Public Health. 2023 Aug 2;3(8):e0001488. doi: 10.1371/journal.pgph.0001488 (PMC10395971; doi:10.1371/journal.pgph.0001488)

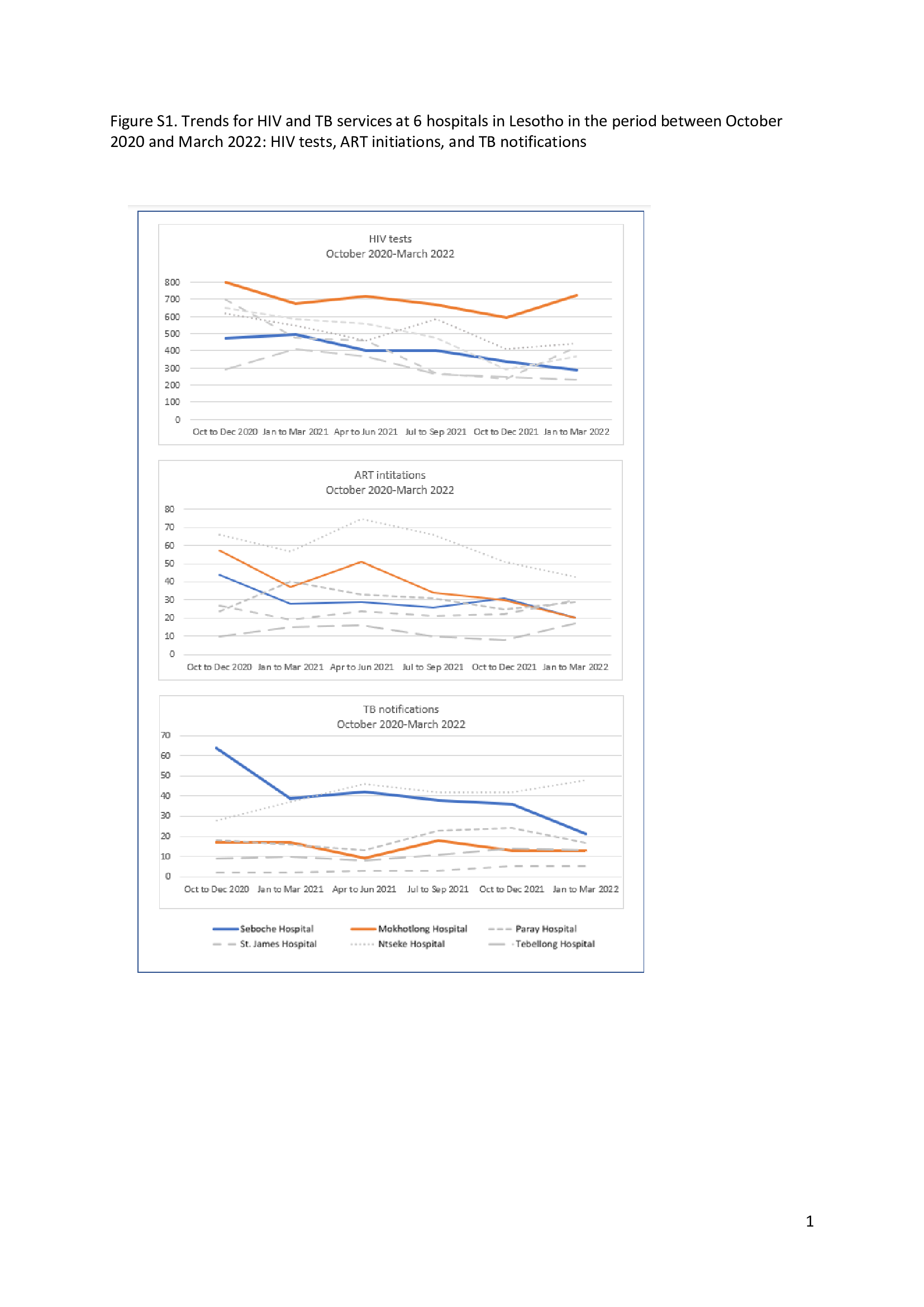

Supplement: S1 Fig — (TIFF) [file pgph.0001488.s001.tiff]
